# Knowledge and acceptability of cervical cancer screening among female undergraduates in Babcock University Ilishan-Remo, Ogun State, Nigeria

**DOI:** 10.3332/ecancer.2023.1502

**Published:** 2023-01-31

**Authors:** Julius O Maitanmi, Temidara E Fabiyi, Oluwadara Eniola, Toluwalope O Sansi, Blessing O Josiah, Bukola Maitanmi, Margaret O Ojewale, Abiodun A Dairo, Damilola A Adebiyi, Oluwadamilare Akingbade

**Affiliations:** 1 School of Nursing, Babcock University, Ilishan Remo, Ogun State 121103, Nigeria; 2 Institute of Nursing Research, Osogbo, Osun State 230262, Nigeria; 3 Turks and Caicos Islands Community College, P.O. Box 236, Grand Turk, Turks and Caicos Islands; 4 Nigerian Army College of Nursing, Lagos 101212, Nigeria; 5 The Nethersole School of Nursing, The Chinese University of Hong Kong, Shatin, Hong Kong 999077, China

**Keywords:** acceptability, cervical cancer, knowledge screening

## Abstract

**Background:**

Cervical cancer (CC) is currently the fourth most common cancer among women. There are several factors which have, in recent times, posed a threat to the recognition and acceptance of CC screening in Nigeria. This study was undertaken to assess the predictors of knowledge of female undergraduates at Babcock University, Ogun State, Nigeria, on CC screening and the level of acceptance readiness.

**Method:**

This study was a descriptive cross-sectional survey of 345 female undergraduates at Babcock University, Ogun State, Nigeria, using a self-administered structured questionnaire. Data collected were analysed using the Statistical Package for the Social Sciences (SPSS) version 23 software. Hypotheses were tested using Pearson product-moment correlation at *p* < 0.05 level of significance.

**Results:**

This study revealed that majority of the respondents have a substantial awareness and knowledge of CC screening (68.4%). The study suggested that health talk and level of exposure might be responsible for the good knowledge. While 47.5% were ready to undergo CC screening, 76.2% were ready to undergo the screening if a health professional performed the procedure, and 66.1% will tell their family members to go for screening. However, 49.9% reported not having the correct information on CC screening. A significant correlation was found between knowledge and acceptability of CC screening among the students (*r* = −0.366, *p* ˂ 0.05). Also, this study suggested that the age of female undergraduates is correlated with acceptance of CC screening (*r* = −0.221, *p* ˂ 0.05).

**Conclusion:**

As the acceptability of CC screening was high, CC screening facilities could be made available for the students. Similarly, educational interventions to improve awareness of CC screening among this population are warranted, as over one-third reported they did not have the correct information on CC screening.

## Introduction

Over the years, cervical cancer (CC), an extant lethal disease, has negatively impacted several women, yet not all women are informed of the possible dangers of not getting screened. Globally, CC is the fourth most common malignancy among women [1]. Annually, Africa records 20% of the new CC cases worldwide, and CC is the leading cause of cancer deaths among females in sub-Saharan Africa [2, 3]. In Nigeria, CC is the second commonest cancer among women of reproductive age and the leading gynaecological malignancy with high mortality among the afflicted [4]. CC accounts for the deaths of over 250,000 women worldwide each year, with an estimated number of 570,000 new cases in 2018, and 80% of these deaths occur in developing and underdeveloped countries [5].

Early screening often detects abnormalities and can be treated before advancing into cancer. Evidence suggests that CC can be cured if diagnosed at an early state and promptly treated [6]. When cancer of the cervix is found early before progressing to the invasive level, the probability of treating it is high [7]. In a study conducted in England, Landy *et al* [8] revealed that the mortality rate of CC would be 5.3 times higher in the absence of screening, while regular screening will make the mortality rate 65% lower. Similarly, various other studies showed that CC screening resulted in a decline in the incidence and mortality rate of CC by up to 90% [9].

Despite the proven benefits of CC screening, Nigeria only has accidental and unevenly distributed CC screening services, reaching less than 9% of women needing the services [10, 11]. In addition to Nigeria’s weak health system and lack of cervical cancer control policy, previous studies have also recognised that lack of awareness, trivialisation of CC, poverty, a low number of female providers, concern for positive screening results and sociocultural norms contribute significantly to poor uptake of CCS services, HPV test and pap smear in Nigeria [12–13].

Women between the age bracket of 30 and 45 are prone to this disease and in some cases, even teenagers, particularly at the age of 18, are likely to be at the mercy of this malignant disease [14]. CC may develop at any age; while it is a lot in women over 40, younger women are also at risk. The risk associated with CC will depend on previous behaviours regarding sex, individual immune system, health status and way of life [15]. Sex debut at an age earlier than 18, having multiple partners or being in relation with persons with various partners increase the possibility of contracting Human Papilloma Virus (HPV) [16].

Furthermore, a study reports that between 50% and 90% of women who die or are diagnosed with CC have not been screened for CC [15]. The most common risk factor for CC is the HPV, which is thought to produce proteins that cause the proliferation of cervical lining cells leading to cancer [17]. However, organised and top-quality cytology-based screening programmes have substantially reduced the burden and risk of CC in various developed countries, even though there were shortcomings in most developing countries like Nigeria [18].

Although Abiodun *et al* [19] noted that the awareness and knowledge of CC and screening were very low, recent data on these variables are limited in this State. Additionally, it is essential to understand the knowledge and acceptability of CC screening and its associated factors among this population, as they are at a high risk of contracting HPV infection [20]. Having this understanding will help develop tailored interventions to address the gaps that might be identified in the knowledge and awareness.

This study was aimed at 1) assessing the level of knowledge of CC screening amongst the female undergraduates at Babcock University, 2) assessing the acceptability of CC screening and 3) understanding factors influencing the acceptability of CC screening among this population. We hypothesised that there is no significant relationship between the ages of female undergraduates at Babcock University.

## Method

### Study design

A descriptive survey was conducted to assess the knowledge and acceptability of CC screening amongst female undergraduates at Babcock University Ilishan, Ogun State, Nigeria.

### Study area

Babcock University is a private faith-based University founded by the Seventh Day Adventist Church in Nigeria. The school is located in Ikenne local government in Ogun State, one of the southwestern Nigerian States.

### Study population

The survey was conducted among female undergraduates of Babcock University at five students’ halls of residence, namely Felicia Adebisi Hall, Queen Esther Hall, Nyberg Hall, White Hall and Havilah Gold Hall.

### Sampling technique

A purposive sampling technique was used to select 345 participants across different age groups, levels, religions, ethnicities and halls of residence.

### Sample size calculation

There were 2,520 female students in the five halls at the time of data collection. The sample size was calculated using the Taro Yamane size formula [21]. *n_o_* = *N*/1+*N*(e)^2^ where: n_0_ is the expected sample size; N is the population size which is 2,520; 1 is the constant; e is the desired level of precision (i.e. the margin of error). We assume we have 95% confidence and 5% precision of error. The value of e is 0.05. *n_o_* = 2520/1+2520(0.05)^2^. = 345.205. Therefore, the sample size was 345.

### Instrument for data collection

The instrument used for the data collection was a self-administered structured questionnaire developed through an extensive literature search. The questionnaire had four sections which include:

**Section A: Measure of demographic characteristics:** Elicited pertinent information about participants’ demographic characteristics (age, school level, religion, ethnicity, marital status and hostel hall).

**Section B: Knowledge of CC screening:** Assessed the knowledge of CC screening among participants. There are 12 questions in all. A score of 1 was allotted to each question. The total was converted to percentage. The knowledge score of participants below 40% was categorised as low, the knowledge score between 40% and 69% was categorised as moderate, while the knowledge score of participants above 70% was categorised as high.

**Section C: Acceptability of CC screening:** Assessed acceptability of CC screening on a 5-point modified Likert scale ranging from Strongly Agree (SA), Agree (A), Undecided (U), Disagree (D) and Strongly Disagree (SD). During analysis, the responses for SA and A were combined as agreed; and SD and D were combined as disagreed.

**Section D: Factors affecting acceptability of CC screening among respondents:** Used to determine the factors affecting acceptability of CC screening on a modified Likert scale. During analysis, the responses for SA and A were combined as agreed; and SD and D were combined as disagreed.

### Validity and reliability of instrument

The face and content validity were ascertained by experts, who ensured the items were relevant and able to elicit required responses in line with the study objectives. Similarly, a reliability test was conducted among 30 female undergraduates at Olabisi Onabanjo University, Sagamu Ogun State. A Cronbach’s alpha of 0.821, 0.700 and 0.744 obtained for the knowledge, acceptability and factors affecting the acceptability of CC screening indicated the instrument’s reliability.

### Data collection

Written and verbal consent was obtained from the respondents. An information sheet which contained detailed information about the research was administered, after which consent was obtained. Confidentiality was assured, as no identifying data was collected, and participation was entirely voluntary. The printed questionnaires were distributed among female undergraduates at Babcock University after a necessary explanation regarding the purpose of the study was provided, and completed copies were collected from the respondents on the same day of administration.

### Data analysis

Data entry was done using Microsoft Excel version 16 and subsequently exported to SPSS version 25. Findings were presented using descriptive statistics such as mean, frequencies and percentages. Hypotheses were tested using the Pearson product-moment correlation at 0.05 level of significance.

### Ethical consideration

Ethical approval for this study was sought and obtained from Babcock University Health Research and Ethical Committee (BUHREC). Verbal and written consent was obtained from the respondents before administering the questionnaires after they were provided information about the study. Participation was voluntary, and confidentiality was assured. All the ethical principles in the Declaration of Helsinki [22] were adhered to.

## Results

Table 1 shows that (7.2%) of the respondents were less than 16 years old and 48.7% were between the ages of 16 and 18 years. The educational level shows 51.0% of the respondents are in 300 level, while 1.7% of them are in 100 level. Furthermore, 76.8% majority of the respondents are Christians. Also, 95.4% were single, while 3.5% were married. Also, the study revealed that 14.2% of the respondents stayed in Queen Esther Hall, while those in Havilah Gold accounted for 25.2%.

As presented in Table 2, 66.1% of the respondents had heard about CC screening and said yes to the question regarding the visual inspection of the cervix as a method of CC screening. Around two-thirds (61.2%) agreed that regular screening prevented CC, and 46.7% thought that CC screening prevents future reproductive problems for the woman.

Meanwhile, 51.3% of them did not know if the use of contraceptives contributes to CC development, and 37.7% of them did not know if cervical screening is done due to complications.

The score was classified into three categories, in which 0–41 (11.3%) was low knowledge, 41–70 (20.3%) was moderate knowledge and 70–100 (68.4%) was high knowledge. The result shows that (68.4%) majority of the respondents had a high knowledge of CC screening.

Table 3 shows that 47.5% of the respondents are ready to do a cervical screening test. The table further shows that 76.2% of the respondents would go for the screening if a healthcare professional performs the procedure, while 66.1% are willing to tell their family members to go for the test. Minority (7.0%) would not recommend others to go for CC screening, and 6.4% were not willing to tell their family members to do a CC screening test.

Table 4 reveals that the majority disagreed that age (62.9%), distance (61.4%), religion (68.2%) and early sexual intercourse (59.4%) reduced their acceptability of CC screening among female undergraduates in Babcock University. 49.9% of the respondents agreed that they did not have the right information on CC screening. Some of the respondents were undecided on the impact of fear (29.5%), expenses (39.4%) or embarrassment during the procedure (28.4%) on their acceptability of CC screening.

### Hypothesis testing

1. There is no significant relationship between the knowledge of CC screening and acceptability among female undergraduates of Babcock University.

Pearson product-moment correlations between the knowledge and acceptability of CC screening among female undergraduates of Babcock University.

Table 5 reveals a significant relationship between the knowledge of CC screening and acceptability among female undergraduates of Babcock University ( −0.366, *p* ˂ 0.05). Therefore, the null hypothesis is rejected.

2. There is no significant relationship between the age of female undergraduate students at Babcock University and the acceptance of CC screening.

Pearson product-moment correlations between the age of respondents and the acceptance of CC screening.

Table 6 shows a significant relationship between the age of female undergraduate students at Babcock University and their acceptance of CC screening. (*r* = −0.221, *p* ˂ 0.05). Therefore, we rejected the null hypothesis (H_0_).

## Discussion

This study aimed to assess the level of knowledge and acceptability of CC screening amongst the female undergraduates at Babcock University. Majority (68.4%) of the respondents had a high level of knowledge of CC screening. Similarly, Neji *et al* [4] evaluated knowledge, attitude and practice of CC screening among female students in tertiary institutions in Calabar and found that 166 students (97.08%) have heard of CC screening, while five students (2.92%) had never heard of CC screening. This is similar to the findings of this study. Their study further reported that 112 participants (65.50%) said that primary prevention of CC is by lifestyle changes and human papillomavirus vaccination, while 59 students (34.50%) had a negative view. Furthermore, Anyebe *et al* [7] examined knowledge and practice of CC screening amongst nurses at Ahmadu Bello University Teaching Hospital Zaria and the study found that 86.7% of their respondents had high knowledge of CC screening; it equally revealed that most of the women have heard about CC screening, and about 78.5% know that CC screening is done by a licensed health professional.

Unlike the current study, which showed a good level of knowledge about cancer screening among respondents, Abiodun *et al* [19], in a similar study conducted in Nigeria, reported an extremely poor level of knowledge. Possible factors responsible for this variation are the sociodemographic characteristics of respondents. Particularly, the respondents in the current study are undergraduates. On the other hand, most (42.4%) of the respondents in the study conducted by Abiodun *et al* [19] had only completed secondary school, which was the highest level of education in the study. Notably, knowledge of CC and Pap smear was significantly lower among outpatients with secondary education [10].

The current study revealed that majority of the respondents had a high level of acceptability of CC screening. This was in tandem with the finding of Ezechi *et al* [23], who also found a high level of CC screening acceptance rate (79.8%), despite a moderate level of awareness of CC and its testing among women who were HIV-positive. Having a tertiary education, having no living child, having a recent HIV diagnosis and awareness of CC were associated with acceptance of CC screening. A cross-sectional descriptive study [24] conducted among 200 female undergraduates in the School of Basic Medical Sciences, University of Benin, showed that 86.7% of the respondents had knowledge of CC and accepted the screening.

Although our results suggest that majority of the respondents have a high level of knowledge of CC screening, 49.9% of the respondents agreed that they did not have the correct information on CC screening. This highlights the need for more interventions to improve the knowledge and awareness of CC screening among this population. This gap in awareness might be one of the factors inhibiting the acceptability of CC screening. It can also be deduced that about 11.6% and 25.5% of the respondents, respectively, strongly agreed and agreed that they were scared of being diagnosed with CC, and a total of 88 respondents making up 25.5% of the respondents strongly disagreed that screening is embarrassing for them. Similarly, a study conducted among Botswana female students on CC screening revealed 315 (94%) attributed CC to smoking and 301 (89.9%) to early sexual debut [25]. The study further revealed that the overall Pap smear screening rate was 92 of 335 students (27.5%). Those who perceived themselves to be at risk of contracting CC were 203 (60.6%) and are 1.8 times more likely to go for a Pap smear than those who perceived it to be safe.

We found a significant relationship between knowledge of CC screening and acceptability among female undergraduates of Babcock University. A systematic review of educational interventions to promote CC screening reported that educational interventions improved the knowledge and uptake of CC screening [26]. Furthermore, we found a significant relationship between the age of the respondents and their acceptance of CC screening. This aligned with the finding of Nene *et al* [27], who also found a significant association between age and acceptance of CC screening.

### Limitation

Although the 345 participants were selected across different age groups, levels, religions, ethnicities and halls of residence; however, the sample might have been prone to researcher bias, which is a limitation of purposive sampling technique. Similarly, this might have affected our ability to generalise the findings from this study. These limitations should be considered when interpreting the findings.

## Conclusion

From this study, it can be concluded that the respondents are knowledgeable about CC screening. Similarly, they demonstrated a high level of acceptability of CC screening. However, a significant proportion were yet to be screened. Therefore, it is important to make screening facilities available for students. Also, more educational interventions are warranted as over one-third of our respondents reported not having the right information on CC screening.

## Conflicts of interest

The authors report no conflicts of interest.

## Funding

No funding was received for this study.

## Figures and Tables

**Table 1.** The demographics data.

Respondents (*N*) = 345			
Demographic	Category	Frequency (*F*)	Percentage (%)
**Age**	Less than 16	25	7.2
	16–18	168	48.7
	19–21	108	31.3
	22–24	40	11.6
	25 and above	4	1.2
**School level**	100	6	1.7
	200	105	30.4
	300	176	51.0
	400	16	4.6
	500	42	12.2
**Religion**	Christians	265	76.8
	Islam	72	20.9
	Traditional	8	2.3
**Ethnicity**	Yoruba	194	56.2
	Igbo	92	26.7
	Hausa	21	6.1
	Others	38	11.0
**Marital status**	Single	329	95.4
	Married	12	3.5
	Divorced/separated	4	1.2
**Hall**	Queen Esther	49	14.2
	Nyberg	63	18.3
	Havilah Gold	87	25.2
	Felicia Adebisi Dada	79	22.9
	White Hall	67	19.4

**Table 2.** Knowledge level of CC screening among female undergraduates in Babcock University.

Variable	Yes Frequency (%)	No Frequency (%)	I don’t know Frequency (%)
Have you heard of CC screening?	228 (66.1%)	109 (31.6%)	8 (2.3%)
Did a health provider talk to you about CC screening?	168 (48.7%)	153 (44.3%)	24 (7.0%)
Is CC screening done by a licensed health professional?	259 (75.1%)	18 (5.2%)	68 (19.7%)
Is CC screening a useful tool for detection of early CC?	245 (71.0%)	16 (4.6%)	84 (24.3%)
Is Pap smear test a test for CC?	156 (45.2%)	64 (18.6%)	125 (36.2%)
Is visual inspection of the cervix a method of CC screening?	137 (39.7%)	28 (8.1%)	180 (52.2%)
CC screening should be done for women ≥ 25 years of age and women with early sex debut or history of multiple sexual partners	113 (32.8%)	42 (12.2%)	190 (5.1%)
Contraceptive use can contribute to CC development	112 (32.5%)	56 (16.2%)	177 (51.3%)
Is CC screening done due to complications?	82 (23.8%)	130 (37.7%)	133 (38.6%)
Regular screening prevents CC	211 (61.2%)	66 (19.1%)	68 (19.7%)
Do you think CC screening can lead to reproductive problem?	106 (30.7%)	106 (30.7%)	133 (38.6)
Do you think CC screening prevents future reproductive problems for the woman?	161 (46.7%)	74 (21.4%)	110 (31.9%)

**Table 3.** Level of acceptability of CC screening among female undergraduates at Babcock University.

Variable	Strongly agree	Agree	Undecided	Disagree	Strongly disagree
I am ready to do a cervical screening test	58 (16.8%)	106 (30.7%)	99 (28.7%)	62 (18.0%)	20 (5.8%)
My reason of taking a CC screening test is to protect myself from disease	80 (23.2%)	138 (40.0%)	79 (22.9%)	44 (12.8%)	4 (1.2%)
I am willing to tell my family members to do a CC screening test	83 (24.1%)	145 (42.0%)	95 (27.5%)	20 (5.8%)	2(0.6%)
I will recommend others to go for CC screening	97 (28.1%)	137 (39.7%)	87 (25.2%)	20 (5.8%)	4 (1.2%)
My religion doesn’t affect my decision to go for CC screening	103 (29.9%)	138 (40.0%)	64 (18.6%)	26 (7.5%)	14 (4.1%)
I would go for screening if a health professional performs the procedure	108 (31.3%)	155 (44.9%)	60 (17.4%)	14 (4.1%)	8 (2.3%)

**Table 4.** Factors influencing acceptability of CC screening among female undergraduates at Babcock University.

Variable	Strongly agree	Agree	Undecided	Disagree	Strongly disagree	Mean score
I am too young to undergo CC screening	12 (3.5%)	48 (13.9%)	68 (19.7%)	117 (33.9%)	100 (29.0%)	2.29
The clinic or hospital is too far for me	18 (5.2%)	49 (14.2%)	66 (19.1%)	96 (27.8%)	116 (33.6%)	2.30
I don’t have the right information on CC screening	51 (14.8%)	121 (35.1%)	48 (13.9%)	73 (21.2%)	52 (15.1%)	3.13
I am scared of being diagnosed with CC	40 (11.6%)	88 (25.5%)	101 (29.5%)	62 (18.0%)	54 (15.7%)	2.99
I feel this screening is embarrassing for me	10 (2.9%)	65 (18.8%)	98 (28.4%)	84 (24.3%)	88 (25.5%)	2.49
My religion doesn’t accept CC screening	8 (2.3%)	38 (11.0%)	64 (18.6%)	133 (38.6%)	102 (29.6%)	2.18
The procedure is too expensive for me	27 (7.8%)	38 (11.0%)	136 (39.4%)	74 (21.4%)	70 (20.3%)	2.65
I think I started having sexual intercourse too early and this may affect the screening	10 (2.9%)	22 (6.4%)	108 (31.3%)	139 (40.3%)	66 (19.1%)	2.34

**Table 5.** Hypothesis one.

			Level of acceptability
Level of knowledge	Pearson correlation	1	−0.366^a^
	Sig. (2-tailed)		0.000
	*N*	345	345

a Correlation is significant at the 0.01 level (2-tailed)

**Table 6.** Hypothesis two.

			Level of knowledge
Age	Pearson correlation	1	−0.221^a^
	Sig. (2-tailed)		0.000
	*N*	345	345

a Correlation is significant at the 0.01 level (2-tailed)
